# Characteristics of motion signal profiles of tonic–clonic, tonic, hyperkinetic, and motor seizures extracted from nocturnal video recordings

**DOI:** 10.1002/epd2.20284

**Published:** 2024-09-16

**Authors:** Petri Ojanen, Csaba Kertész, Jukka Peltola

**Affiliations:** ^1^ Faculty of Medicine and Health Technology Tampere University Tampere Finland; ^2^ Neuro Event Labs Tampere Finland; ^3^ Department of Neurology Tampere University Hospital Tampere Finland

**Keywords:** epilepsy, motor seizures, signal analysis

## Abstract

**Objective:**

In this study, characteristics of signal profiles formed by motion, oscillation, and sound signals were analyzed to evaluate generalizability and variability in a single patient setting (intra‐patient variability) and between patients (inter‐patient variability). As a secondary objective, the effect of brivaracetam intervention on signal profiles was explored.

**Methods:**

Patient data included 13 hyperkinetic seizures, 65 tonic seizures, 13 tonic–clonic seizures, and 138 motor seizures from 11 patients. All patients underwent an 8‐week monitoring, and after a 3‐week baseline, brivaracetam was initiated. Motion, oscillation, and sound features extracted from the video were used to form signal profiles. Variance of signals was calculated, and combined median and quartile visualizations were used to visualize the results. Similarly, the effect of intervention was visualized.

**Results:**

Hyperkinetic motion signals showed a rapid increase in motion and sound signals without oscillations and achieved low intra‐patient variance. Tonic component created a recognizable peak in motion signal typical for tonic and tonic–clonic seizures. For tonic seizures, inter‐patient variance was low. Motor signal profiles were varying, and they did not form a generalizable signal profile. Visually recognizable changes were observed in the signal profiles of two patients.

**Significance:**

Video‐based motion signal analysis enabled the extraction of motion features characteristic for different motor seizure types which might be useful in further development of this system. Tonic component formed a recognizable seizure signature in the motion signal. Hyperkinetic and motor seizures may have not only significantly different motion signal amplitude but also overlapping signal profile characteristics which might hamper their automatic differentiation. Motion signals might be useful in the assessment of movement intensity changes to evaluate the treatment effect. Further research is needed to test generalizability and to increase reliability of the results.

**Key points**

- Signal profiles formed by motion, oscillation, and sound signals presented different characteristics for different motor seizure types.
- Drug intervention showed changes in motion signals which might be useful in the assessment of treatment effect.
- Further studies are required to examine generalizability of signal profiles in different seizure types.

## 1 INTRODUCTION

According to previous research, even 30% of epilepsy patients suffer from drug‐resistant epilepsy (DRE)^1^ which significantly increases mortality, morbidity, and sudden unexplained death in epilepsy (SUDEP).^2^, ^3^ Also, DRE reduces quality of life of patients, but, however, patients tend to tolerate uncontrolled seizures and symptoms related to them better than adverse effects of anti‐seizure medications (ASMs).^4^ When balancing between quality of life of patients and possible side effects of ASMs, seizure severity and SUDEP risk should be evaluated clinically. Some older studies have reported symptom‐based questionnaires of seizure severity scales^5^, ^6^ but, according to our knowledge, they do not relate to neuronal effects in EEG, and relation to SUDEP risk has not been evaluated which weakens the utility of those scales in clinical practice. To the best of our knowledge, quantitative methods to analyze seizure severity have not been reported.

As previously reported, approximately 50% of daytime seizures, and even 80% nocturnal seizures are missed by patients or caregivers when a seizure diary is used as a documentation method.^7^, ^8^ Patients and their caregivers might not be able to document and objectively assess the seizure symptoms and their severity,^9^ which makes the seizure documentation and evaluation of severity change difficult within drug interventions. The inaccuracies of seizure diaries can significantly affect the intervention outcome assessment in clinical practice and within drug trials, and the difference of documentation information between seizure diaries and video monitoring can lead to a different conclusion on treatment effect.^10^ Also, variability of seizure duration and movement intensity in an intra‐patient and inter‐patient setting might remain unnoticed due to poor documentation. In a previous study, a video monitoring system was able to recognize the change in movement intensity before and after a drug intervention^11^ but variation of movement intensity between seizures has not been explored. New seizure documentation and alarm methods, such as video monitoring systems, have improved seizure documentation and classification, and enabled better evaluation of treatment efficacy.^12^, ^13^

Video monitoring systems have previously utilized movement quantification to detect seizures. Movement signals have been reported in detection of myoclonic, clonic, and random movements of neonatal patients by using motion‐strength and motion activity signals.^14^ Another study formed motion signatures from motion signals of the face and hands to analyze the seizure semiology.^15^ Also, accelerometers and multimodal systems have been explored to analyze movements.^16^, ^17^, ^18^ However, the generalizability of these motion signals is limited, and as most studies focus on the performance of seizure detection, the generalizability of motion signals for different seizure types has not been examined.

The Nelli seizure monitoring system is an audio/video‐based semi‐automatic (hybrid) seizure monitoring system which utilizes computer vision and machine learning to analyze kinematic data of motion, oscillation, and audio that are related to seizures with positive motor component and human experts to visually evaluate the video epochs.^11^ It has been previously reported to detect motor seizures with promising accuracy, and to reduce workload from annotation and seizure classification.^19^ According to a recent study, the seizure movement signals were successfully utilized for automatic seizure classification.^20^ Despite the potential of the system to detect changes in movement intensity and seizure duration, quantitative analysis has not been examined. Also, we think that utilizing signal profiles could help further studies of automatic seizure classification and assessment of seizure intensity change.

In this study, our primary object is to further investigate the seizure signal profiles first presented in a previous case study.^21^ We study DRE patients with motor seizures who underwent 8‐week home monitoring and brivaracetam intervention. Our aim is to analyze signal profiles including motion, oscillation, and sound signals to assess their utility for further development of seizure detection and classification and to explore the intra‐patient and inter‐patient variability of signal profiles within a seizure type to evaluate the generalizability of signal profiles. The signal profiles are also compared before and after the intervention to determine whether movement intensity changes can be visually evaluated from motion, oscillation, and sound signal figures.

## 2 METHODS

### 2.1 Patient population

Overall, 15 patients with focal DRE were enrolled to study, and 11 of them experienced seizures with motor manifestations during the monitoring period and thus were included in the analysis. Six of the patients participated in a previous seizure classification study.^20^ The study protocol and informed consent forms were approved by the Ethics Committee of Tampere University Hospital. Signed informed consent was obtained from patients prior to participation in this study. All patients used two or more ASMs, and some of them were also treated with vagal nerve stimulation (VNS) therapy.

In this study, patients underwent 8‐week home monitoring in which BRV was initiated at the beginning of the fourth week. Each patient was monitored for 7–13 h per night (average 9.8 h, median 9.5 h). Video monitoring data were first reviewed by expert annotators, who marked all video events that were suspected to be seizures. Those suspected seizure events were then confirmed to be epileptic by neurologists by comparing them to previous video‐EEG monitoring (VEM) reports, and unequivocality of seizure semiology was then determined based on the VEM reports which is a feasible reference standard for the phase two study.^22^ Only unequivocal seizures confirmed to be epileptic were included in the analysis. Seizures were classified by professionals according to ILAE 2017 guidelines.^23^ Patients with VNS were monitored if VNS was activated during seizures. The seizure semiology was determined based on the ictal manifestations in the video data. Patient demographics have been presented in Table 1.

**TABLE 1:** Patient demographics: Age, seizure type and semiology, ASMs used with daily dose in parenthesis.

Patient ID	Age	Seizure type	Seizure semiology	ASM (daily dose)
1	29	FIAMS (I.B.01)	Motor BL—Vocalization	N.A.
2	30	FHS (I.C.08)	Hyperkinetic BL—Vocalization	N.A.
3	36	FIAMS (I.B.01)	Motor BL—Facial contraction BL—Vocalization	Lacosamide (600), Topiramate (500), VNS
4	37	FIAMS (I.B.01)	Eyes open—Motor BL	Lamotrigine (550), Lacosamide (400), VNS
			Facial contraction BL—Tonic R—Motor BL	
5	61	FIAMS (I.B.01)	Behavior arrest—Head version L—Motor L	N.A.
6	19	GTCS (II.A.09)	Eyes open—Tonic BL—Clonic BL—Change in breathing	Lamotrigine (150), Levetiracetam (250), Clobazam (5), Valproate (1200)
		FIATS (I.B.04)	Eyes open—Tonic BL—Change in breathing	
7	35	FIAH (I.B.07)	Eyes open—Hyperkinetic activity—Vocalization	Eslicarbazepine (1600), Pregabalin (450), VNS
8	39	N.A.	Eyes open—Tonic BL—Facial contraction BL—Clonic BL	Lamotrigine (500), Zonisamide (300), VNS
		FIAMS (I.B.01)	Eyes open—Motor BL—Change in breathing	
9	40	FTS (I.C.05)	Eyes open – Tonic BL—Change in breathing	Lamotrigine (400), Topiramate (200), VNS
			Eyes open—Tonic BL—Clonic BL	
10	18	FMS (I.C.01)	Eyes open—Facial contraction BL—Motor BL	Oxcarbazepine (900), Lacosamide (200)
11	18	FTS (I.C.05)	Eyes open—Tonic BL	Valproate (1200), Lamotrigine (200), Topiramate (200), Clobazam (25), VNS
		FMS (I.C.01)	Eyes open—Tonic BL—Vocalization—Restless movement	

Abbreviations: BL, bilateral; FHS, focal hyperkinetic seizure; FIAH, focal impaired awareness hyperkinetic seizure; FIAMS, focal impaired awareness motor seizures; FIATS, focal impaired awareness tonic seizure; FMS, focal motor seizure; FTS, focal tonic seizure; GTCS, generalized tonic–clonic seizure; L, left; N.A., information not available; R, right; VNS, vagus nerve stimulation.

### 2.2 Video monitoring

Video monitoring was performed by utilizing the Nelli® seizure monitoring system developed by Neuro Event Labs, Tampere, Finland. The system consists of a camera and a microphone installed at the bedside at the patient's home so that the patient stays in sight of the camera during periods of rest, for example, during nighttime. The software included in the system enabled analysis of the recorded video. Video data were cropped from the raw data by a professional epileptologist in order to decrease the background noise of the video event and to optimize the seizure signal analysis. Video clips were cropped so that they included the seizure onset and the assumed ending of the seizure activity by comparing VEM reports and the seizure manifestations in the video. Preictal and postictal phases were left out from the analysis.

### 2.3 Signal profiles

The method to extract motion and oscillation features and to create time‐series signal data were described previously in detail.^21^ Similarly, time‐series data of motion and oscillation were utilized in this study.

A background subtraction method^24^ was combined with a stereo correspondence filter.^25^ The background subtraction model then formed a binary mask of the moving pixels of the frames, and the ratio of moving pixels and all pixels in an image determined a one‐dimensional signal for a video. For the oscillation signal, an optical flow‐based method was used. A time‐series motion vector field was calculated to form a motion path history, where only the unbroken paths during a 1‐s time period were analyzed for direction reversal (a reversal is considered a change of direction over 90°). Also, 2.5 Hz oscillation frequency has been found useful in separation of ictal oscillation.^20^, ^21^

Similarly, as in the proof‐of‐concept study,^21^ signal profiles were formed to present motion, oscillation, and sound signals of each seizure type and each patient in a column. Variance of signals was calculated for each seizure semiology. By comparing signal profile figures and variances of each seizure semiology of each patient and between patients, the intra‐patient and inter‐patient variability of seizures and seizure types could be assessed. To improve the evaluation of intra‐patient variability of seizures, the most representative seizure for each seizure semiology for each patient was chosen to act as a reference for other seizures. The most representative seizure was chosen by an expert epileptologist based on the visual analysis of typical and distinct ictal manifestations on the video with minimal background noise and artifacts from lighting and blanket. Combined median and quartile visualizations were then utilized based on the created motion, oscillation, and sound signals to improve readability and visual analysis.

To visualize the effect of brivaracetam intervention, combined median and quartile visualizations were separately used for seizures before and after the intervention for each seizure type. Also, mean signal values (which were averaged over the seizure duration) and their variance were calculated. Based on mean signal values and the visually recognizable changes in motion signals, the effect of intervention could be assessed.

## 3 RESULTS

### 3.1 Seizures during monitoring

Our dataset consisted of 138 motor seizures, 13 hyperkinetic seizures, 65 tonic seizures, and 13 tonic–clonic seizures. Table 2 shows seizure counts for each patient from the baseline period before intervention and from the follow‐up period. All patients experienced seizures during the baseline monitoring, while only eight patients had seizures during the follow‐up. One patient (patient 6) discontinued the brivaracetam without experiencing seizures during the follow‐up. Six patients had VNS but only one patient was able to activate the device (in 9 of 10 seizures).

**TABLE 2:** Seizures during monitoring, and before and after intervention for each patient.

Patient number	Seizures during monitoring	Seizures before intervention	Seizures after intervention	VNS activation rate
1	16 M	6	16	‐
2	2 HK	2	0	‐
3	37 M	12	25	0/37
4	10 M	8	2	9/10
5	28 M	12	16	‐
6	11 TC, 9 T	11 TC, 9 T	0	‐
7	11 HK	3	8	0/11
8	2 TC, 31 M	2 TC, 18 M	13 M	0/33
9	33 T	22	11	0/33
10	5 M	5	0	‐
11	23 T, 11 M	12 T, 4 M	11 T, 7 M	0/34

*Note*: VNS activation column: ‐, patient does not have VNS.

Abbreviations: HK, hyperkinetic; M, motor; T, tonic; TC, tonic–clonic.

### 3.2 Signal profiles of seizures

For each patient and seizure type, combined median and quartile visualizations were created based on signals of motion, oscillation, and sound, and they were organized in the same column to represent seizure signal profile. The most representative seizure has been bolded black in motion, oscillation, and sound signals; and the median of seizures were colored blue. Variance was colored light blue shading in all figures. Based on the visual analysis of signals and their variance, the variability of seizures could be assessed. In our dataset, patients experienced hyperkinetic, tonic–clonic, tonic, and motor seizures. Original seizure signals which the median and quartile visualizations were calculated from have been presented in Supplementary Materials.

**FIGURE 1:**
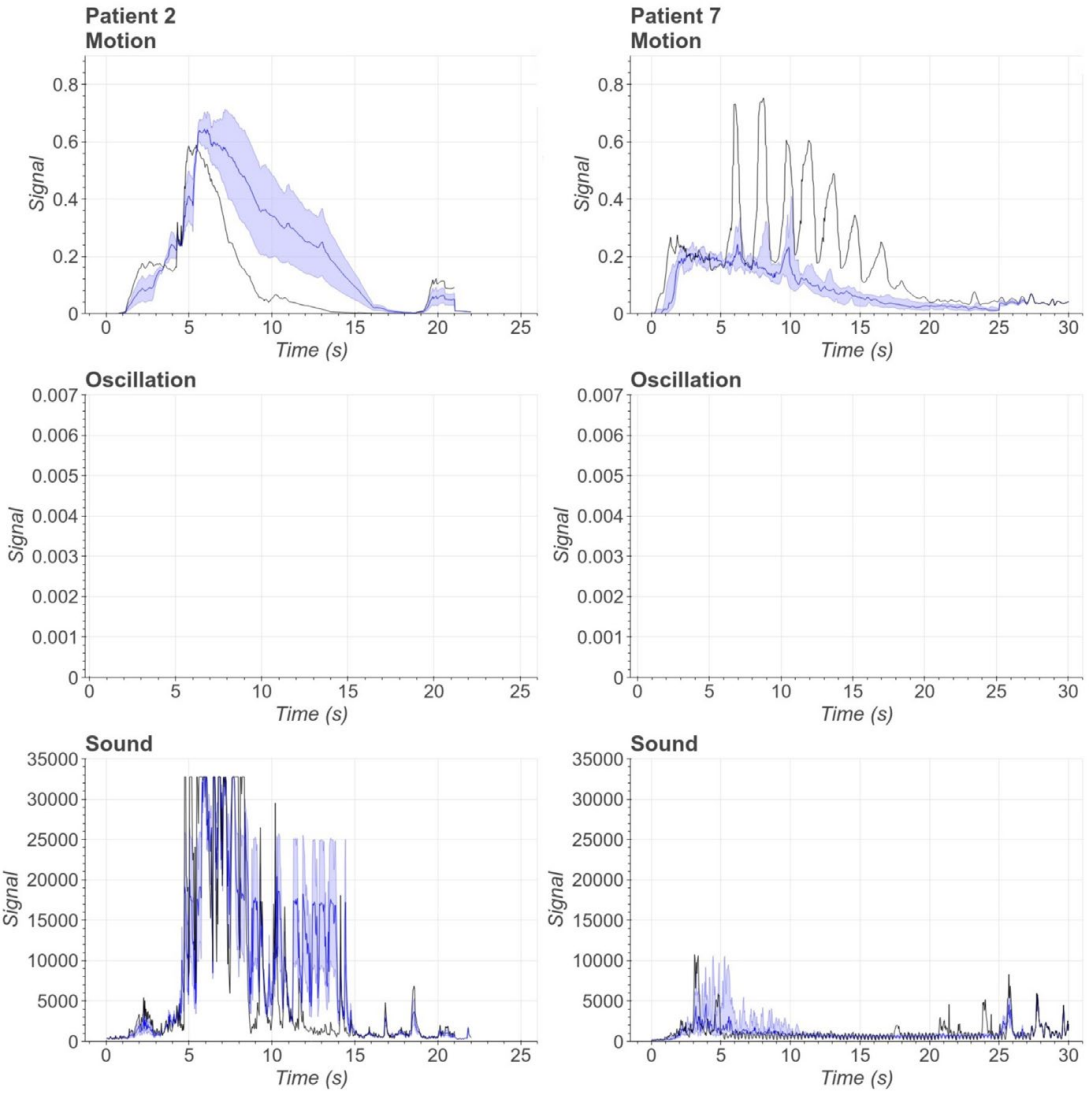
Combined median and quartile visualisations for signal profile figures of hyperkinetic seizures from 2 patients. The most representative seizure has been bolded in all signal figures.

Figure 1 shows hyperkinetic seizure signal profiles from two patients. Both seizures manifest an abrupt increase in motion signal which is caused by sudden movement in the recorded video. However, after the start of the seizures, there are high spikes in the motion signal after 5–10 s from the onset. That is likely caused by patients' limbs that move close to the camera during the given time period, as the motion signal increases especially when moving objects are close to the camera. These peaks increase the motion signal to significantly higher values than in other seizure types. Patient 2 fell from bed during both seizures and kicked the camera which also affected the motion signal. The oscillation signal remained low during the hyperkinetic phase because ictal manifestations such as kicking motion did not reach the threshold frequency of 2.5 Hz. Sound signal activity was caused by kicking and hitting the bed in both patients but also the mattress alarm increased the sound in patient 7. Variance was small especially in seizures of patient 7, and shape of the motion signal was similar for both patients, as well as absence of oscillation.

**FIGURE 2:**
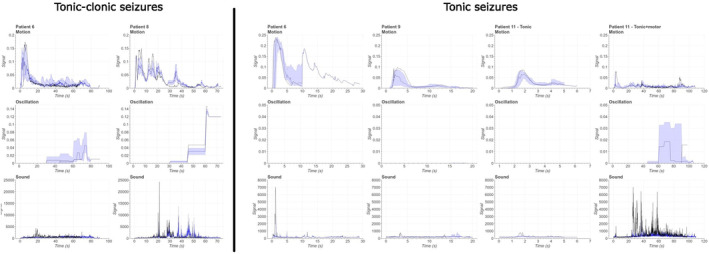
Combined median and quartile visualisation for signal profile figures of tonic–clonic seizures from 2 patients and tonic seizures from 4 patients. The most representative seizure of both seizure types has been bolded in all signal figures.

Figure 2 presents tonic–clonic seizure signal profiles from two patients on the left, and tonic seizure signal profiles from four patients on the right. The sudden increase in motion signal shows a quick onset of tonic activity in both seizure types, and the tonic phase seems to manifest a spike form. Patient 6 had one tonic seizure with individual clonic movements after the tonic phase which caused two spikes in the motion signal. Oscillation does not exist in the tonic seizures of patients 6 and 11. Patient 9 had increased oscillation signal after the motion spike which was caused by a short clonic component after the tonic phase in one seizure. However, the clonic phase was short and the seizure was not considered a tonic–clonic seizure. Other seizures did not have an increase in oscillation signal (see original signals from Supplementary Materials). Patient 11 had seizures with a tonic component in two different seizure semiologies: tonic seizures have only a short tonic activity, but motor seizures had a restless motor activity with head turning, kicking, and arm movements after the tonic phase which caused oscillation activity. These seizures were marked as “tonic” and “tonic + motor,” respectively, in Figure 2.

Regarding the tonic–clonic seizures, patient 8 had a longer motion signal spike during the tonic phase which was caused by individual clonic movements before the start of clonic phase and the movement of caregiver in sight of camera. Clonic phase of tonic–clonic seizures increases the oscillation signal, and the oscillation increases after the tonic spike in motion signal which may indicate the ability to distinguish tonic and clonic phases. In patient 8, oscillation seems to rise at the end of the seizure despite the end of clonic activity even though there was no oscillating movement in sight of the video at a given time point. Tonic–clonic seizures have similar motion and oscillation signal shapes; a sudden motion increase following a phase with oscillation activity. Inter‐patient variance of motion signal was significant in tonic and tonic–clonic seizures, even though intra‐patient variance was relatively small in these seizure types.

**FIGURE 3:**
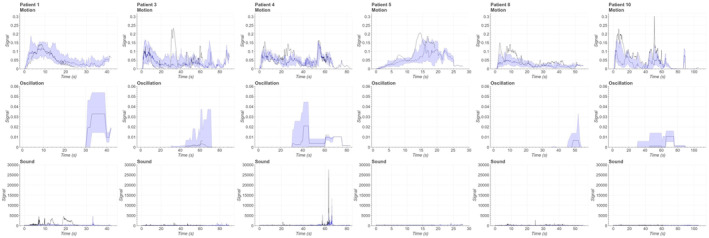
Combined median and quartile visualisations for signal profile figures of motor seizures from 6 patients. The most representative seizure has been bolded in all signal figures.

Signal profiles of motor seizures have been presented in Figure 3. As these motor seizures were not further classified, this category included seizures with different manifestations. Variance of motion and oscillation signal was distinguishable in both intra‐patient and inter‐patient settings. Onset can be varying depending on the patient, and the motion signal may have one or multiple spikes during the seizure. Also, the oscillation and sound activity vary a lot between seizures in both intra‐patient and inter‐patient settings. Unlike in the previous seizure types, motor seizures do not form any generalizable form or other distinctive signal characteristics, as expected. However, some of these signal profiles may mimic signal profiles from other seizure types, as signal profiles from patients 1, 3, and 10 may be falsely interpreted to be tonic–clonic seizures. On the other hand, according to VEM reports, motor seizures of patient 8 had the same epileptic activity only in the beginning as tonic–clonic seizures without generalization, which may explain the similar signal manifestations of these seizure semiologies.

### 3.3 Effect of intervention on signal profiles

**FIGURE 4:**
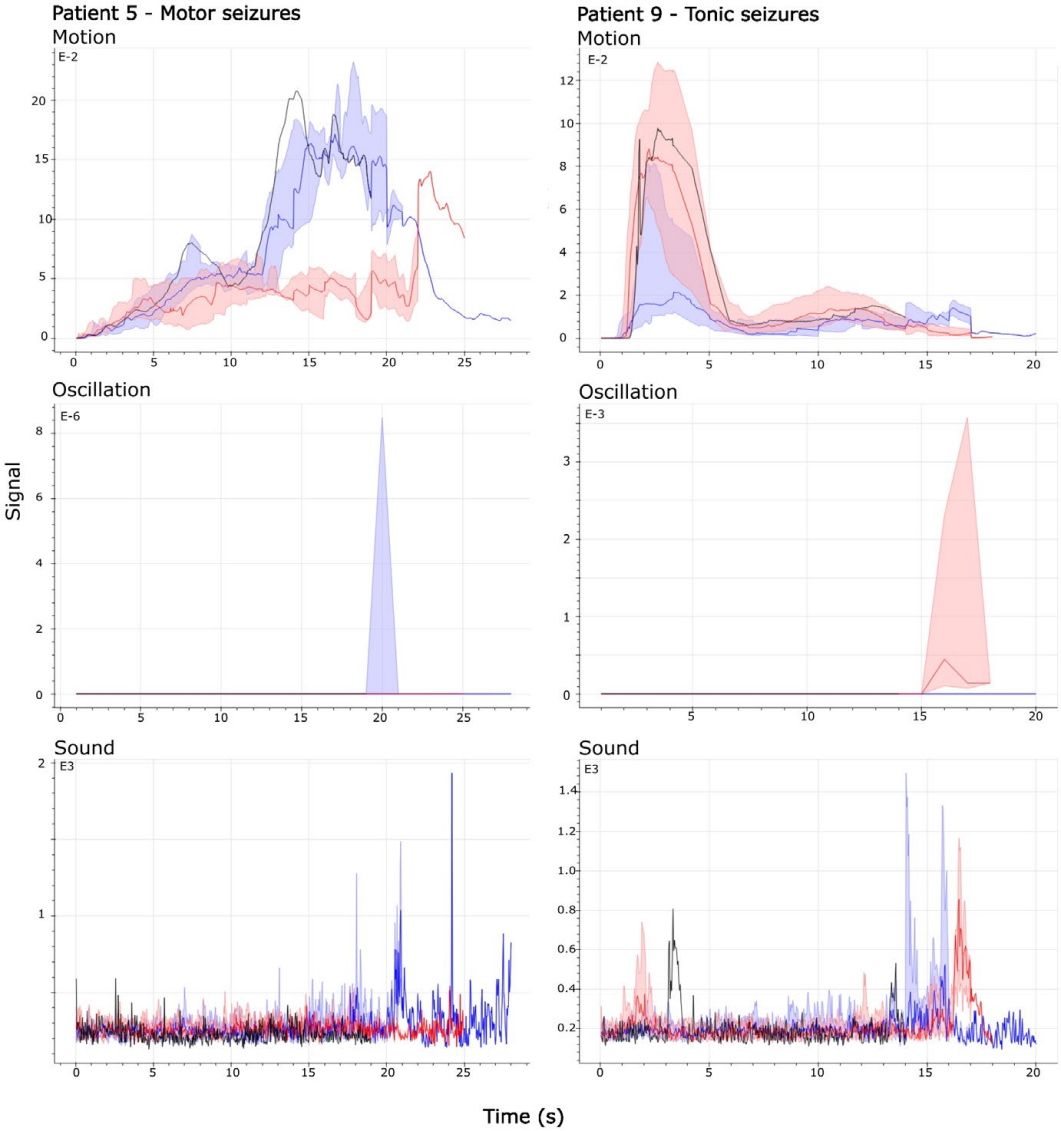
Combined median and quartile visualisations for two patients with significant changes in their signal profiles. Blue and red lines depict median values of signals, and blue and red shadings depict the variance of these signals, before and after the intervention, respectively.

From 11 patients who participated in this study, 10 patients underwent the treatment through follow‐up successfully and eight patients experienced seizures both before and after the intervention. Of eight patients, only patients 5 and 9 had visually recognizable changes in their signal profiles which have been presented in Figure 4. The changes were confirmed by mean signal values of these patients, as patient 5 experienced 36% and 7% decrease in mean motion and sound values, while patient 9 experienced 61% and 7% increase in those signals, respectively. Also, patient 4 experienced 5%, 47%, and 33% decrease, and patient 11 experienced 13%, 0%, and 7% decrease in motion, oscillation, and sound signals, respectively. However, based on the visual analysis of figures and video data, there are no clinically relevant changes in these patients. Median visualizations and mean signal values of motion, oscillation, and sound of other patients before and after the intervention have been presented in Supplementary Materials.

Figure 4 presents the median visualization of seizures before and after the intervention. Median and variance of signals before intervention were marked with a blue signal and blue shading, while red signal and red shading indicate median and variance of seizures after the intervention, respectively. As in the previous figures, the most representative seizures were marked with a black signal. As shown in Figure 4, patient 5 has lower motion and oscillation signal values after the treatment, especially after 12 s from the onset. Patient 9, however, had higher motion signal values after the treatment, even though the variance of signals was high. Thus, patient 5 experienced a decrease and patient 9 experienced increased movement intensity after the initiation of brivaracetam.

## 4 DISCUSSION

In this study, our intention was to investigate further the signal profiles in a larger and more diverse patient population. Hyperkinetic seizures have a rapid onset in motion signal in both patients, and higher motion signal values with the absence of oscillation activity may separate them from other seizure types. Tonic–clonic seizures as well as tonic seizures had similarities in their signal profiles of tonic phase, and clonic oscillation can be utilized to separate the two seizure types. Especially hyperkinetic and tonic–clonic seizures had repetitive, stereotypical findings in their motion signals with small variance. Furthermore, the significant effect of brivaracetam intervention on signal profiles was detected in two patients.

In a recent study, tonic and hyperkinetic seizures were automatically separated with high accuracy but the classification of tonic–clonic seizures was challenging possibly due to an unbalanced dataset.^20^ Signal profile patterns found in this study may explain the previous findings, as tonic seizures often had similar signal manifestations throughout the dataset which may indicate more accurate recognition of the tonic component. The oscillation of the clonic phase combined with the tonic motion manifestation may be useful to distinguish tonic–clonic seizures from other seizure types. In this study, semiological characteristics were varying which manifested in the signal profiles, especially in motor seizures, which may have reduced the generalizability of signal profiles. If two seizure types with the same onset have varying propagation, it might hamper the separation of those seizure types in a video dataset of a single patient. Quantitative analysis and recognition of specific moving body parts by utilizing pose estimation during seizures could be a solution to this problem but, according to our knowledge, functional models have not been reported in a home setting.^26^

In previous literature, motion analysis has been utilized in seizure detection, and several studies have shown seizure signal characteristics typical for a given seizure type. For example, clonic seizures have been represented by detecting movement patterns with luminance signals which created a rhythmic signal with multiple peaks in even distribution distinguishable from normal motions.^27^ Similarly, clonic movement has been separated from normal movement with optical flow methods which resulted in different signals of horizontal movements.^28^, ^29^ Hyperkinetic seizures have also been separated from automotor seizures with high probability.^30^ Sound has been utilized in semiology analysis,^31^ and seizure detection by utilizing threshold selection.^32^ In order to separate oscillations (clonic component) from other movements, we utilized an optical flow‐based algorithm which seemed to recognize clonic components of tonic–clonic seizures, even though oscillation signals increased also in seizures without clonic movements. According to this dataset, the sound signal may help to distinguish major motor features, such as hyperkinetic and tonic–clonic seizures from unspecific motor seizures. Tonic phase was recognized in this larger patient population similarly as in the previous study^21^ but other studies related to motion analysis of tonic or unspecific motor seizures were not found. Inter‐ or intra‐patient motion signal variance of motor seizures has not been reported before.

As previously noticed, video monitoring has many advantages compared to seizure diaries during drug interventions. In comparison to seizure diaries, video monitoring has been shown to improve seizure documentation during drug interventions and enable analysis of motor manifestations.^11^ In this study, we noticed significant changes in motion signals before and after the intervention in two patients. Changes in motion manifestations of seizures can hamper seizure documentation which might be challenging for patients and their caregivers. Furthermore, the activation of VNS during monitoring was observed. Only one of six patients activated VNS during seizures which indicates the inability to achieve benefits from the device for most of the patients in this study population. Video monitoring might help clinicians to evaluate a patient's ability to activate VNS during seizures.

In this study, the amount of hyperkinetic and tonic–clonic seizures was low which may limit the generalizability of the signal profiles and make visualizations of medians of signals more prone to the effect of background noise and other artifacts. Due to the availability, we only included hyperkinetic, tonic, tonic–clonic, and motor seizures which may have more typical and distinguishable movement manifestations. However, the semiology of seizures within seizure types can significantly affect the signal profiles, especially in the motor seizure category, which may decrease the generalizability of the signal profiles of motor seizure type. Thus, the methodology presented in this study cannot be considered more reliable for seizure classification compared to human observations. Movements of caregivers in sight of the camera can also affect seizure signal profiles by causing artifacts which may reduce the reliability and thus generalizability of signal profiles. However, movements of other people are challenging to filter out from the video recorded in a home setting. Change in lighting conditions may affect signals, and a blanket may impede the small movements of patients. Also, we did not include non‐epileptic events which might have had similar motion figures as seizures presented in this study. On the other hand, high accuracy of this system to automatically detect various seizure types from non‐epileptic events has been reported.^19^ Even though seizures with subtle motor manifestations have been detected with high accuracy,^33^ analyzing their motion signals might be more challenging than signals of seizures with major motor symptoms. Due to the variation of seizure duration, the quartile and median visualizations rely on a smaller amount of seizure data towards the end of signals which may increase the risk of inaccuracies. On the other hand, original seizure signal profiles provided in the Supplementary Materials are not affected by this issue. The 5‐week follow‐up period in this study is relatively short, and thus the changes in motion signals before and after intervention may not be visible yet for all patients. In addition, due to the absence of EEG in this study, the relation between motion signal changes and seizure intensity decrease is not confirmed.

## 5 CONCLUSIONS

The video‐based motion signal analysis enabled extraction of motion features characteristic of different motor seizure types which might be useful in seizure classification and further development of this system. Tonic motion component has a motion signal signature typical for tonic and tonic–clonic seizures. Hyperkinetic and motor seizures could be differentiated based on the amplitude of the motion signal but there are also overlapping motion signal profiles which may cause challenges for the task. Motion signals also showed visually recognizable changes after the drug intervention which might be utilized in the evaluation of treatment effect. Further research with a more evenly distributed dataset and more seizure types are still needed to improve the reliability and usability of seizure signal profiles.

## CONFLICT OF INTEREST STATEMENT

CK is an employee of Neuro Event Labs, the company that provided the equipment and technology used in the study. PO has provided medical consultation for Neuro Event Labs. JP is a shareholder of Neuro Event Labs. JP has participated in clinical trials for Eisai, UCB, and Bial; received grants from Eisai, Medtronic, UCB, and Liva‐Nova; received speaker honoraria from LivaNova, Eisai, Medtronic, Orion Pharma, and UCB; received support for travel to congresses from LivaNova, Eisai, Medtronic, and UCB; and participated in advisory boards for Arvelle, Novartis, LivaNova, Eisai, Medtronic, UCB, and Pfizer. All authors contributed to the interpretation of data and revision of the manuscript.

## Supporting information


Data S1.
