# Supplementary material for: Characteristics of motion signal profiles of tonic–clonic, tonic, hyperkinetic, and motor seizures extracted from nocturnal video recordings
Source: Epileptic Disord. 2024 Sep 16;26(6):804–13. doi: 10.1002/epd2.20284 (PMC11651380; doi:10.1002/epd2.20284)
Supplement: Supplementary file 1 — Data S1. [file EPD2-26-804-s001.docx]

Supplementary material

Original seizure signal profiles


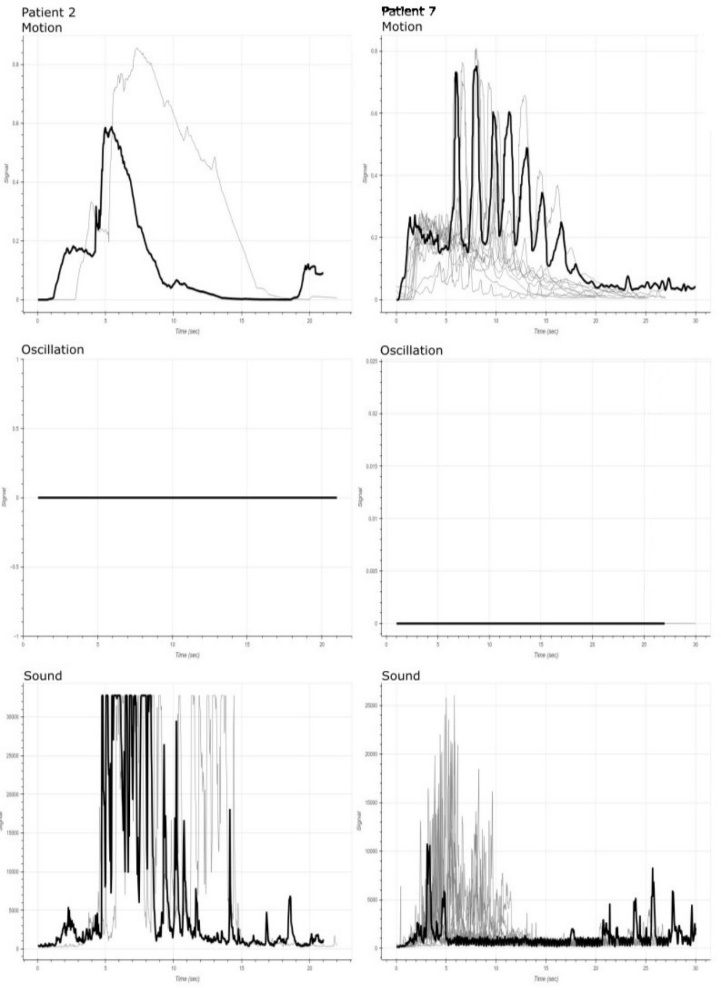


Supplementary material, figure 1. Hyperkinetic seizure signal profiles.


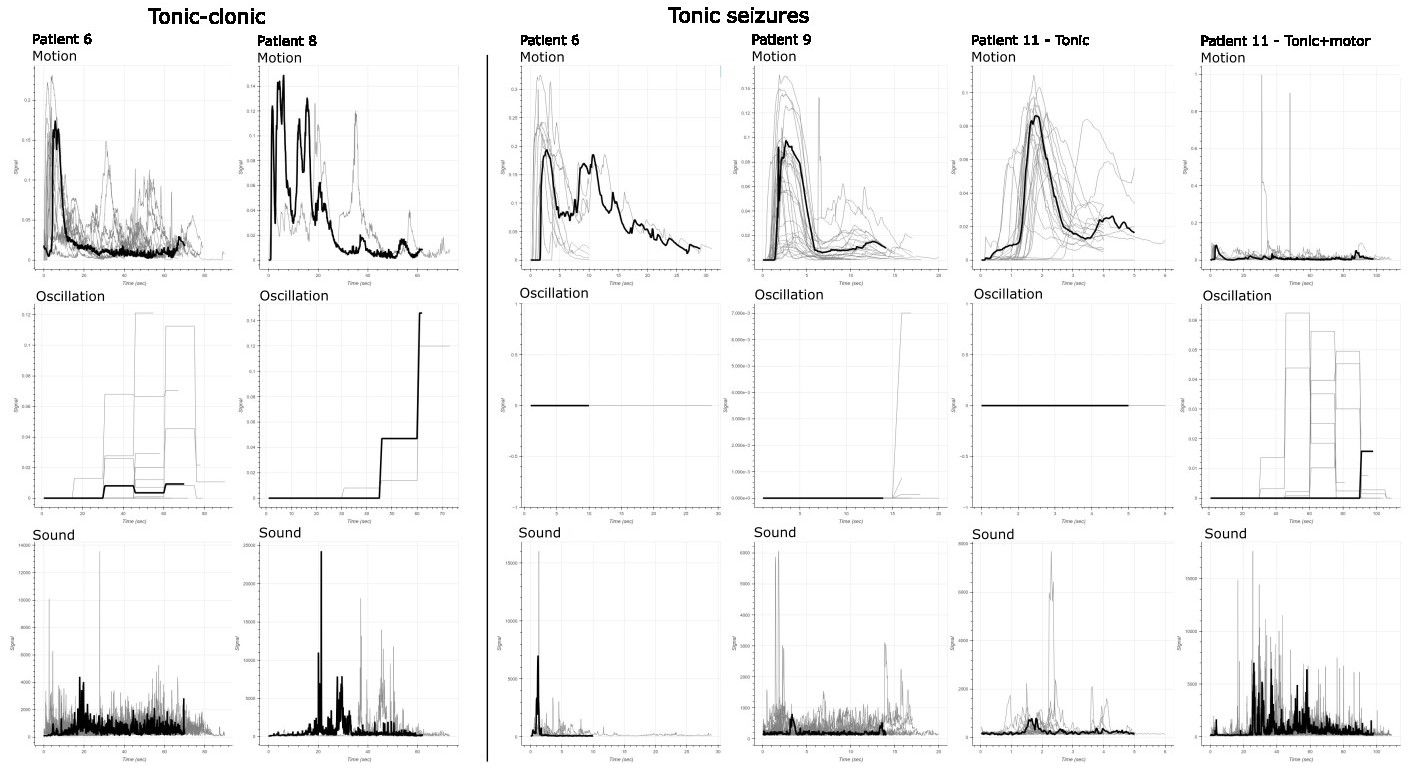


Supplementary material, figure 2. Tonic-clonic and tonic seizure signal profiles.


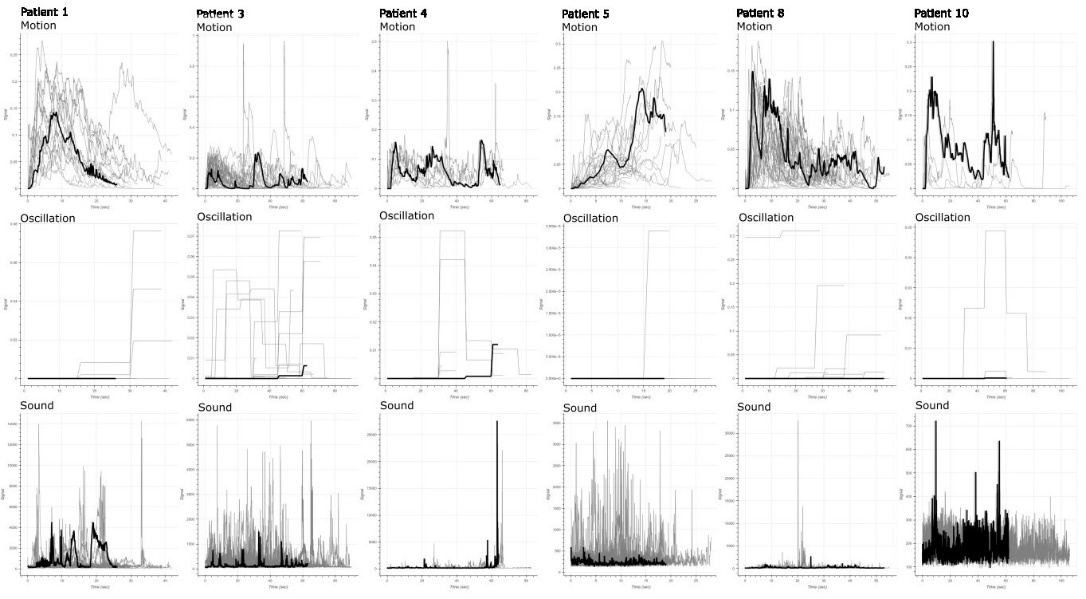


Supplementary material, figure 3. Motor seizure signal profiles.


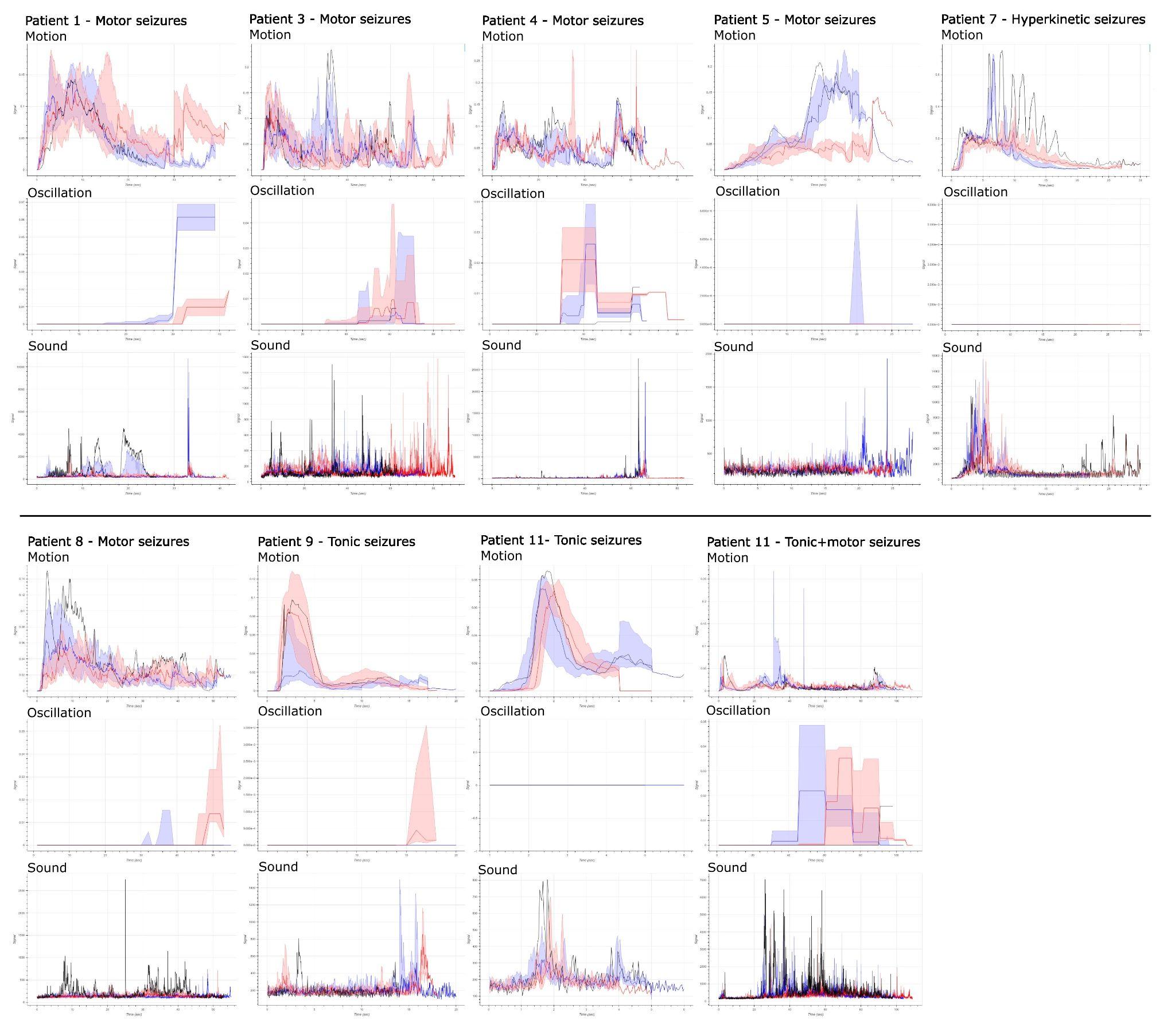
 Supplementary material, figure 4. Intervention signal profiles from all patients who underwent brivaracetam intervention.

Supplementary material, table 1. Mean signal values of each patient before and after the brivaracetam intervention, variances presented in parentheses. *N.A = Not Available

| Patient | Motion | | Oscillation | | Sound | |
| --- | --- | --- | --- | --- | --- | --- |
|  | Before (Variance) | After (Variance) | Before (Variance) | After (Variance) | Before (Variance) | After (Variance) |
| 1 | 0.0465 (0.016) | 0.0633 (0.0236) | 0.0145 (0.0019) | 0.0025 (0.001) | 289.812 (106.3645) | 240.6611 (73.8857) |
| 3 | 0.038 (0.019) | 0.0349 (0.0199) | 0.0005 (0.0004) | 0.0013 (0.0012) | 125.0814 (28.1013) | 121.9689 (31.1567) |
| 4 | 0.0481 (0.0154) | 0.0456 (0.0084) | 0.0037 (0.0019) | 0.007 (0.0025) | 361.349 (64.3296) | 239.6151 (44.266) |
| 5 | 0.0657 (0.0124) | 0.0421 (0.0105) | 0.0 (0.0) | 0.0 (0.0) | 280.9905 (37.3122) | 259.248 (35.5217) |
| 7 | 0.0993 (0.0162) | 0.0813 (0.0183) | 0.0 (0.0) | 0.0 (0.0) | 1121.0242 (272.8371) | 938.7844 (309.9089) |
| 8 - motor | 0.024 (0.012) | 0.0214 (0.0108) | 0.0 (0.0) | 0.0013 (0.0005) | 134.4394 (21.4597) | 137.2682 (25.7524) |
| 9 | 0.0072 (0.0031) | 0.0186 (0.0092) | 0.0 (0.0) | 0.0 (0.0) | 196.0625 (31.5179) | 211.1472 (39.1796) |
| 11 - tonic | 0.0204 (0.008) | 0.0176 (0.0054) | 0.0 (0.0) | N.A (N.A) | 189.7194 (25.1819) | 176.3767 (22.285) |
| 11 - tonic+ motor | 0.0086 (0.003) | 0.009 (0.0042) | 0.0056 (0.0045) | 0.0056 (0.0053) | 346.158 (82.8714) | 315.1765 (83.0934) |
